# Gating of Quantum Interference in Molecular Junctions by Heteroatom Substitution

**DOI:** 10.1002/anie.201609051

**Published:** 2016-11-29

**Authors:** Xunshan Liu, Sara Sangtarash, David Reber, Dan Zhang, Hatef Sadeghi, Jia Shi, Zong‐Yuan Xiao, Wenjing Hong, Colin J. Lambert, Shi‐Xia Liu

**Affiliations:** ^1^ Department of Chemistry and BiochemistryUniversity of BernFreiestrasse 33012BernSwitzerland; ^2^ Quantum Technology CentrePhysics DepartmentLancaster UniversityLancasterLA1 4YBUK; ^3^ Collaborative Innovation Center of Chemistry for Energy MaterialsDepartment of Chemical and Biochemical EngineeringCollege of Chemistry and Chemical EngineeringXiamen UniversityXiamen361005China

**Keywords:** density functional calculations, heteroatom effects, molecular electronics, quantum interference, single-molecule transport

## Abstract

To guide the choice of future synthetic targets for single‐molecule electronics, qualitative design rules are needed, which describe the effect of modifying chemical structure. Here the effect of heteroatom substitution on destructive quantum interference (QI) in single‐molecule junctions is, for the first time experimentally addressed by investigating the conductance change when a “parent” meta‐phenylene ethylene‐type oligomer (m‐OPE) is modified to yield a “daughter” by inserting one nitrogen atom into the m‐OPE core. We find that if the substituted nitrogen is in a meta position relative to both acetylene linkers, the daughter conductance remains as low as the parent. However, if the substituted nitrogen is in an ortho position relative to one acetylene linker and a para position relative to the other, destructive QI is alleviated and the daughter conductance is high. This behavior contrasts with that of a para‐connected parent, whose conductance is unaffected by heteroatom substitution. These experimental findings are rationalized by transport calculations and also agree with recent “magic ratio rules”, which capture the role of connectivity in determining the electrical conductance of such parents and daughters.

Recent experiments demonstrating room temperature quantum interference (QI) in single‐molecule junctions have stimulated intense interest in the development of molecular switches,^1^ transistors^2^ and thermoelectric devices^3^ with improved performance. Much effort has been devoted to controlling QI effects by modifying electronic structures or molecular topologies.2b, 4 However, a direct correlation between QI and chemical structure remains ambiguous. For example, destructive QI has been observed in linearly conjugated molecules, while constructive QI occurs in cross‐conjugated molecules of the close‐loop type.^5^ Amongst all molecules investigated to date, nonalternant and alternant hydrocarbon systems have been intensively investigated.^6^ Although heterocyclic aromatics are particularly interesting due to the presence of spatially separated conductance pathways, which offer opportunities for varying QI, only a few papers about the effect of heteroatoms on single‐molecule junction conductances have appeared in the literature.^7^ Recently, we developed a new magic ratio rule (MRR) showing that the conductance ratio of two molecules through the same aromatic core with different connectivties is equal to the square of the ratio of their magic integers.^8^ The accuracy of the MRR for polycyclic aromatic hydrocarbons (PAHs) is demonstrated in Ref. 8b, in which MRR predictions for the ratios of conductances for a range of PAHs are compared with experiments. A remarkable feature of the MRR is that the associated most‐probable conductance ratios are independent of the binding geometry, elongation etc., even though the conductances themselves do depend on such features. As discussed in Ref. 7c and in the Supporting Information (SI), this insensitivity can be expected, provided the statistical distribution of contact geometries is independent of connectivity. A generalization of this theory7c that predicts three simple rules governing the effect of heteroatom substitution in alternate hydrocarbon parents (see also SI), has not yet been verified experimentally. As a consequence, we set ourselves the task of investigating how destructive QI in the “parent” *meta*‐phenylene ethylene‐type molecule (***m*** **‐OPE**) changes when one nitrogen atom is inserted into the central benzene ring at different positions, to yield the “daughter” molecules **M1**, **M2** and **M3** in Scheme 1. To assess the heteroatom effect on QI and to gain insight into the correlation between connectivity and electrical conductance, the parent *para*‐OPE (***p*** **‐OPE**) and its daughter molecule **P** (Scheme 1) were also studied.

**Scheme 1:**
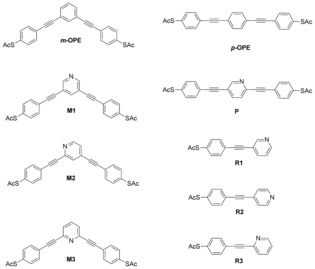
Chemical structures of the investigated molecules.

The target compounds ***m*** **‐OPE**, ***p*** **‐OPE**, **P** and **M1**–**M3** were synthesized via Sonogashira cross‐coupling reactions of *S*‐4‐iodophenyl ethanethioate with the corresponding diethynylbenzene/pyridine. All of them were purified using column chromatography and characterized by NMR and high‐resolution mass spectrometry (see SI). Charge transport measurements of single‐molecule junctions with molecules shown in Scheme 1 were performed using a mechanically controllable break junction (MCBJ).^9^ Figure 1 A displays typical conductance (*G*) versus distance (Δ*z*) stretching traces, as plotted in a semi‐logarithmic scale. The single‐molecule conductance plateaus of 6 molecules vary from 10^−3.5^ to 10^−6^ *G*_0_ besides the plateau presenting the gold–gold atomic contact located at *G*_0_, while the blank experiment using THF/TMB solvent without molecules shows no single‐molecule plateau.

**Figure 1:**
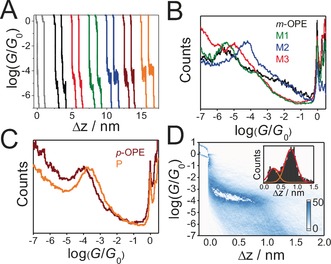
A) Typical individual conductance–distance traces of blank experiment in TMB/THF (gray) and molecules ***m*** **‐OPE** (black), ***p*** **‐OPE** (brown), **P** (orange), **M1** (green), **M2** (blue) and **M3** (red). B,C) All‐data‐point 1D conductance histograms constructed from 1000 MCBJ traces of each molecule. D) All‐data‐point 2D conductance versus relative distance (Δ*z*) of the molecule **P**. The stretching distance distribution is determined from the range of 0.7 *G*_0_ to 10^−5^ *G*_0_.

Figure 1 B,C show the 1D conductance histograms of molecules, constructed from 1000 experimental conductance–distance traces for each compound without data selection. After the rupture of gold–gold atomic contacts at *G*_0_, a single conductance peak, corresponding to the most probable conductance for each molecule was detected (also summarized in Table 1). Briefly, the conductance values follow the trend **M2**>**M3**>**M1**=***m*** **‐OPE**, suggesting that heteroatom substitution leads to an increase in conductance only when the substituted nitrogen is *ortho* to both contacts or *para* to one connection and *ortho* to the other. On the other hand, there is a negligible change in electrical conductance when the nitrogen is *meta* to both contacts, in agreement with Ref. 7c. As depicted in Figure 1 C, the presence of the nitrogen atom shows no distinguishable effect on the constructive QI in the junctions through the *para*‐coupled molecules ***p*** **‐OPE** and **P**, which is in good agreement with Ref. 7c and the results obtained by Gonzalez et al.7b

The two‐dimensional (2D) conductance versus distance histogram of molecule **P** demonstrates a clear molecular density cloud at around 10^−4.0^ *G*_0_ (Figure 1 D), which is quite similar to that of ***p*** **‐OPE**.6c Interestingly, there is no significant conductance feature of the molecular junctions with one of Au electrodes connected directly to the central pyridine ring via the Au−N bonds, as observed in the analogous system containing a pyrimidine ring.7b This observation can be attributed to the significant binding force difference between the Au−S covalent bond (1.2 nN)^10^ and the Au−N bond (0.8 nN)^11^ that is close to the Au−S (methylsulfide) bond (0.7 nN),^12^ leading to a preference for the covalent Au−S over the Au−N binding motif in the present case. Furthermore, the conductance plateau is ca. 0.8 nm (after adding the snap back distance of 0.5 nm it will be 1.3 nm), suggesting that the most pronouncing feature stems from the molecules trapped only via Au−S bonds. However, the 2D histograms of the *meta*‐coupled molecules (Figure S9, SI) show that the conductance cloud becomes less clear as previously reported in the literature.4d, 13 Apparently, the bent molecular geometry strongly affects the evolution of molecular junctions, resulting in a slanted conductance region. Thus, it is impossible to plot the plateau‐length distribution and further calculate the molecular length in the *meta*‐coupled systems.

To further verify our hypothesis of an end‐to‐end configuration via the Au−S binding motif and to completely rule out the possibility of the conductance plateaus being due to the molecules bonded to one of the electrodes through the N atom at the central ring, we have performed control experiments of the reference compounds **R1**–**R3** containing only one SAc group (Figure 2).

**Figure 2:**
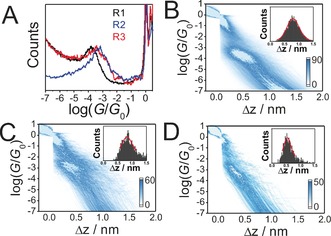
A) 1D conductance histograms of **R1** (black), **R2** (blue) and **R3** (red), constructed from 1000 MCBJ traces of each molecule. B–D) 2D conductance histograms of **R1**–**R3**, respectively, and stretching distance Δ*z* distributions (inset). The stretching distance distribution is determined from the range of 0.7 *G*_0_ to 10^−5^ *G*_0_.

All of them provide clear evidence for creation of molecular junctions with distinctly higher conductance values compared to those of **M1**–**M3**. They are arranged in the following order of their decreasing conductance, the *para*‐coupled molecule **R2** (10^−3.2±0.1^ *G*_0_) > the *ortho*‐coupled molecule **R3** (10^−3.6±0.1^ *G*_0_) > the *meta*‐coupled molecule **R1** (10^−3.9±0.1^ *G*_0_), which is a clear signature of the QI effect. As illustrated in Figure 2 B–D, a clear intensity cloud is observed for each case and the plateau length for **R2** is longer than for **R1** and **R3**. The most probable stretched distance Δz* is determined from the plateau distribution histograms to be 0.8 nm (**R2**), 0.7 nm (**R1**) and 0.6 nm (**R3**), respectively. As a results, the most probable absolute distance z* (z*=Δz* + 0.5 nm)9c between two gold tips is 1.3 nm for **R2**, 1.2 nm for **R1** and 1.1 nm for **R3**, which agrees well with theoretically determined junction lengths (1.16 nm for **R2** 1.09 nm for **R1** and 0.96 nm for **R3**, Table S3). It can therefore be deduced that the distinct variations in conductance values of **M1**–**M3** and **R1**–**R3** are attributed to the different electronic transport pathways whereby the former molecules are trapped between two Au leads via the Au−S bonds while the latter ones are connected on the one side via the Au−S bonds, on the other side via the Au−N bonds. All these results provide direct evidence for an unambiguous preference for the covalent Au−S over the Au−N binding motif in the junctions through **M1**–**M3**, which verifies the proposed junction configurations.

The above heteroatom effect can be understood by applying the theory of Ref. 7c, which identifies the ratio of two molecular conductances with the ratio of two “energy‐dependent core transmission functions”, evaluated at the Fermi energy (see SI). As discussed in section 4 of the SI, these energy‐dependent core transmission functions are proportional to the Green's function of the core of the molecule and are independent of the nature of the coupling to the electrodes.

For the three‐ring molecules of Scheme 1, the energy dependences of the core electron transmission coefficients are shown in Figure 3 A. For ***m*** **‐OPE**, the parental M‐function and the corresponding core transmission function vanish at *E*=0, due to destructive QI in the middle of the HOMO–LUMO gap. However, by adding a heteroatom to the central benzene ring, core transmission coefficients of the molecules **M2** and **M3** become non‐zero while the core transmission coefficient of the molecule **M1** remains zero at *E*=0. On the other hand, the transmission functions of *para*‐coupled systems ***p*** **‐OPE** and **P** are non‐zero at *E*=0 and overlap with each other, indicating that the heteroatom effect is negligible in the constructive QI systems. Illustrated in Figure 3 B are the full electron transmission coefficients obtained from DFT mean field Hamiltonian.^14^, ^15^

**Figure 3:**
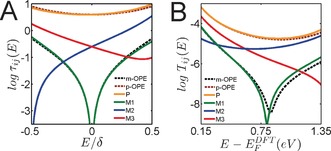
A) Core transmission coefficients *τ*_ij_(*E*) of the molecules ***m*** **‐OPE**, ***p*** **‐OPE**, **P**, **M1**–**M3** against *E*/*δ*, where *δ* is half of the HOMO–LUMO gap of the parental core; i.e. *δ*=1, using DFT transport approach implemented in Gollum.^15^ B) The calculated transmission coefficients *T*_ij_(*E*) of the molecules ***m*** **‐OPE**, ***p*** **‐OPE**, **P**, **M1**–**M3**, connected to gold electrodes using the mean‐field Hamiltonian from Siesta.^14^ Dashed lines correspond to “parents” and solid lines to “daughters”.

Table 1 shows the experimental values for the most probable conductances of the molecules ***m*** **‐OPE,** ***p*** **‐OPE, P and M1**–**M3**, which are divided by conductance value of molecule **M3**. Comparison with the corresponding core‐transmission ratios of Figure 3 A also reveals that the best agreement with experimental data is obtained with a daughter Fermi energy of *E*_F_^d^=0.211, which differs from the mid‐gap of the daughter (*E*_HL_^d^=−0.0795) and parent (*E*_HL_^p^=0), indicating the variation of the alignment of intrinsic molecular orbital (MO) levels due to heteroatom substitution. Although the lone‐pair electrons of the N atom are held in an sp^2^ hybrid orbital perpendicular to the orbitals in the π‐system, its presence has an unambiguous effect on the energy levels and nodal properties of the MOs (Figure S6, SI). Both experiment and DFT‐based theory reveal that the presence of the nitrogen atom shows no discernible effect on the constructive quantum interference in the junctions through the *para*‐coupled molecules as a result of the fact that the core transmissions are only slowly varying functions of energy. In the absence of a Fermi energy shift, the ratio of conductances for **M1** and **M3** should vanish. Taking a shift of *E*_F_^d^=0.211 into consideration, M‐theory yields a conductance ratio of 0.37 that compares well with the experimental ratio of 0.32. The same holds true for the conductance ratio of **M2**/**M3**. All of these results are captured by the minimal theory,7c which identifies the conductance ratios with the square of the ratio of two “magic numbers” (Table S2, SI).

**Table 1:** A summary of an experimental and theoretical study on the single‐molecule junction conductances of ***m*** **‐OPE**, ***p*** **‐OPE**, **P** and **M1**–**M3**.

Molecule	Experimental conductance	Conductance ratio^[a]^	DFT conductance ratio^[b]^	Core transmission	Core transmission ratio^[e]^
***m‐*** **OPE**	10^−5.5±0.05^	0.32	0.02	0^[c]^	0
***p‐*** **OPE**	10^−4.0±0.1^	12.59	13.40	4^[c]^	30.77
**P**	10^−4.0±0.1^	12.59	14.00	4.75^[d]^	36.6
**M1**	10^−5.55±0.05^	0.32	0.04	0.048^[d]^	0.37
**M2**	10^−4.35±0.05^	5.18	5.12	0.67^[d]^	5.15
**M3**	10^−5.1±0.05^	1	1	0.13^[d]^	1

[a] Experimental conductance divided by experimental conductance of **M3**. [b] DFT conductance divided by DFT conductance of **M3** at *E*_F_=0.9. [c] Core transmission of parents at *E*_F_^p^=0. [d] Core transmission of daughters at *E*_F_^d^=0.211. [e] Core transmission divided by core transmission of **M3**.

In conclusion, it has been demonstrated that the destructive QI can be alleviated by the heteroatom substitution whereas constructive QI is almost unaffected. The increase in conductance for *meta*‐coupled OPEs originates from two distinct effects. First, the change in the Hamiltonian modifies the interference pattern within the core, the energy dependence of the associated M‐functions and core transmission coefficients. Secondly, heteroatom substitution shifts the Fermi energy relative to the parent, which is also analyzed in the MO viewpoint. Our results provide a qualitative guide for tuning QI effect in single‐molecule junctions that is of prime importance in designing molecular devices with desirable functions.

## Supporting information

As a service to our authors and readers, this journal provides supporting information supplied by the authors. Such materials are peer reviewed and may be re‐organized for online delivery, but are not copy‐edited or typeset. Technical support issues arising from supporting information (other than missing files) should be addressed to the authors.

Supplementary
