# Supplementary material for: Gating of Quantum Interference in Molecular Junctions by Heteroatom Substitution
Source: Angew Chem Int Ed Engl. 2016 Nov 29;56(1):173–6. doi: 10.1002/anie.201609051 (PMC5396325; doi:10.1002/anie.201609051)
Supplement: Supplementary file 1 — Supplementary [file ANIE-56-173-s001.pdf]

Angewandte Chemie
Eine Zeitschrift der Gesellschaft Deutscher Chemiker

Supporting Information

Gating of Quantum Interference in Molecular Junctions by Heteroatom Substitution

Xunshan Liu+, Sara Sangtarash+, David Reber+, Dan Zhang+, Hatef Sadeghi, Jia Shi, Zong-Yuan Xiao, Wenjing Hong,* Colin J. Lambert,* and Shi-Xia Liu**

anie_201609051_sm_miscellaneous_information.pdf

1. Synthesis and characterization of the target compounds

The synthetic routes of the pyridine derivatives **P**, **M1-M3**, and the benzene derivatives ***m*-OPE** and ***p*-OPE** are shown in Scheme **S1**. 2,4-dibromopyridine is commercially available from Sigma-Aldrich. 1,3-Diethynylbenzene,[1] *S*-4-iodophenyl ethanethioate,[2] 1,4-diethynylbenzene,[3] 3,5-diethynylpyridine,[4] 2,5-diethynylpyridine,[5] 2,6-diethynylpyridine,[6] (4-thioacetylphenyl)(3-pyridyl)acetylene (**R1**),[7] (4-thioacetylphenyl)(4-pyridyl)acetylene (**R2**)[8] and (4-thioacetylphenyl)(2-pyridyl)acetylene (**R3**)[9] were synthesized according to published procedures. All target compounds and intermediates have been fully characterized. Their NMR spectroscopic and high-resolution mass spectrometric data are consistent with their proposed structures. ^{1}H and ^{13}C NMR spectra were recorded on a Bruker Avance spectrometer at 300 and 75.5 MHz, respectively. Chemical shifts are reported in parts per million (ppm) and are referenced to the residual solvent peak ($CDCl_3$, ^{1}H = 7.26 ppm; ^{13}C = 77 ppm). Coupling constants (J) are given in hertz (Hz) and are quoted to the nearest 0.5 Hz. Peak multiplicities are described in the following way: s, singlet; d, doublet; t, triplet; m, multiplet. HRMS data was obtained with ESI (electrospray ionization) mode. The detailed synthetic processes are as follows.

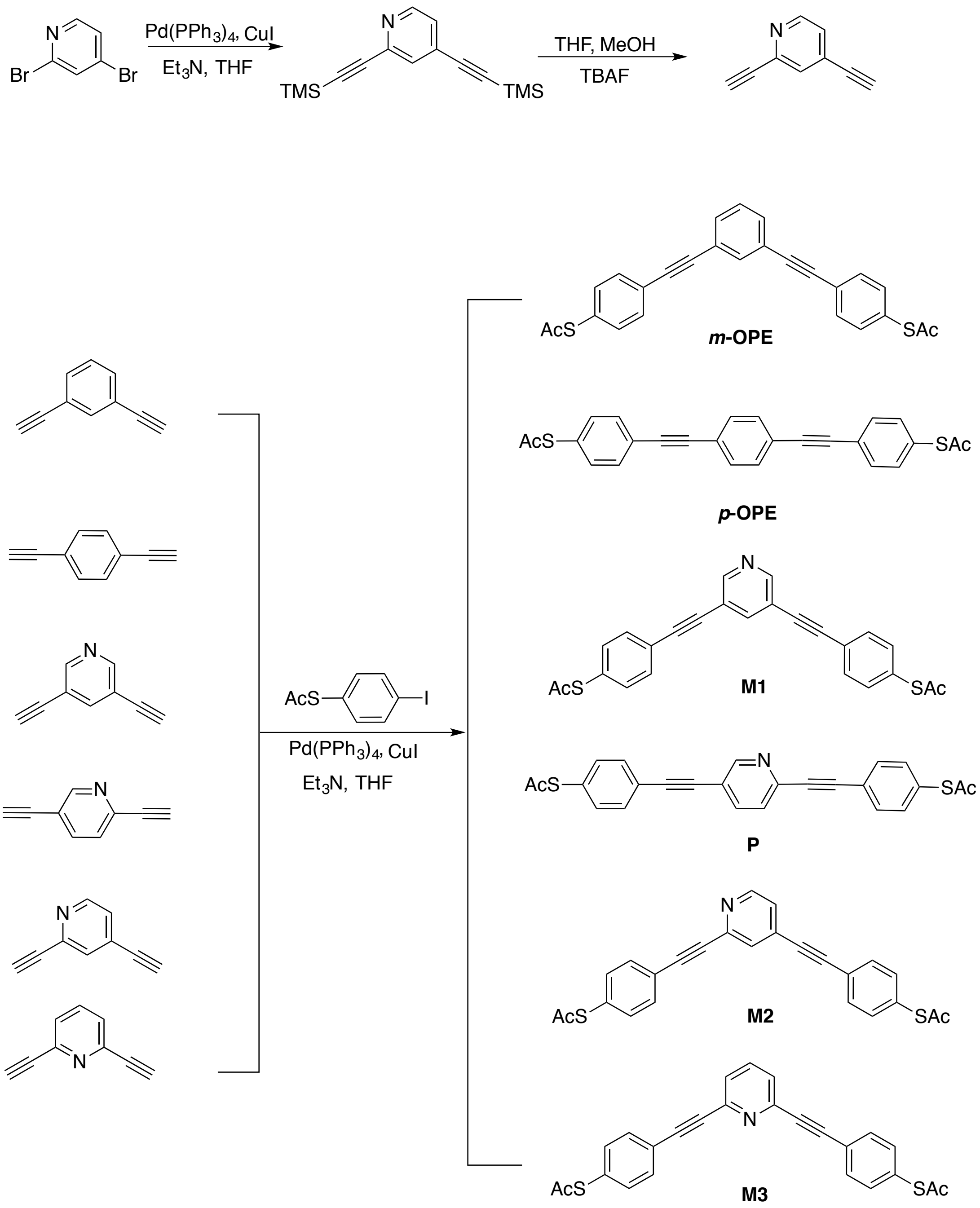

Scheme S1 Synthetic routes to target compounds.

Synthesis of 2,4-bis(2-(trimethylsilyl)ethynyl)pyridine. 2,4-Dibromopyridine (200 mg, 0.85 mmol), ethynyltrimethylsilane (175 mg, 1.79 mmol) CuI (20 mg, 0.11 mmol) and $Pd(PPh_3)_4$ (80 mg, 0.07 mmol) were mixed in a solution of Et_3N (1 mL) and dry THF (3 mL). After degassing for 15 minutes, the mixture was stirred at 80 °C for 6 h in microwave. After cooling to room temperature, the solvent was removed in vacuo. The crude product was purified on silica gel chromatography using a hexane/dichloromethane mixture (3/1 by volume) as eluent to afford the analytically

pure product. Yield: 155 mg (67%); ^{1}H NMR (300 MHz, $CDCl_3$): δ 8.51-8.49 (dd, J = 0.8 Hz, J = 5.10 Hz, 1H), 7.50-7.49 (m, 1H), 7.23-7.21 (dd, J = 1.5 Hz, J = 5.10 Hz, 1H), 0.26 (s, 9H), 0.25 (s, 9H); HRMS (ESI): *m/z* calcd for $C_{15}H_{22}NSi_2$: 272.1285; found: 272.1280 ($M+H^+$).

Synthesis of 2,4-diethynylpyridine. Under nitrogen atmosphere, 2,4-bis(2-(trimethylsilyl)ethynyl)pyridine (109 mg, 0.40 mmol) and 0.08 mL MeOH were dissolved in dry THF (4 mL). At 0 °C, tetrabutylammonium fluoride (1.0 M in THF, 0.2 mL, 0.2 mmol) was added dropwise and the resultant solution was stirred for 15 min at 0 °C. Then all solvents were removed under reduced pressure, the resultant residue was purified by column chromatography on silica gel using dichloromethane/hexane = 1:2 (v/v) as eluent to afford the analytically pure product. Yield: 48 mg (94%); ^{1}H NMR (300 MHz, $CDCl_3$): δ 8.57-8.55 (dd, J = 0.8 Hz, J = 5.1 Hz, 1H), 7.54-7.53 (t, J = 2.0 Hz, 1H), 7.33-7.31 (dd, J = 1.5 Hz, J = 5.1 Hz, 1H), 3.32 (s, 1H), 3.18 (s, 1H); HRMS (ESI): *m/z* calcd for C_9H_6N: 128.0495; found: 128.0492 ($M+H^+$).

Synthesis of *m*-OPE. To a solution of 1,3-diethynylbenzene (20 mg, 0.16 mmol) and *S*-4-iodophenyl ethanethioate (92 mg, 0.33 mmol) in THF (3 mL) were added Et_3N (0.5 mL) and the catalysts $Pd(PPh_3)_4$ (20 mg, 0.017 mmol) and copper iodide (0.005 g, 0.025 mmol). The reaction mixture was stirred for 6 h in microwave at 50 °C. The solvent was removed under reduced pressure, and the residue was purified by silica gel flash chromatography using a hexane/dichloromethane mixture (1/1 by volume) as eluent to provide the desired compound. Yield: 34 mg (51%); ^{1}H NMR (300 MHz, $CDCl_3$): δ 7.72-7.71 (t, J = 2.8 Hz, 1H), 7.57-7.55 (d, J = 8.4 Hz, 4H), 7.51-7.49 (dd, J = 1.5 Hz, J = 7.4 Hz, 2H), 7.42-7.39 (d, J = 8.4 Hz, 4H), 7.35-7.33 (dd, J = 0.8 Hz, J =4.9 Hz, 1H), 2.44 (s, 6H); ^{13}C NMR (75.5 MHz, $CDCl_3$): δ 193.4, 134.7, 134.3, 132.2, 131.6, 128.6, 128.3, 124.2, 123.4, 90.1, 89.3, 30.3; HRMS (ESI): *m/z* calcd for $C_{26}H_{19}O_2S_2$: 427.0821; found: 427.0812 ($M+H^+$).

Synthesis of *p*-OPE. Following the same synthetic procedure for ***m*-OPE**, 1,3-diethynylbenzene was replaced with 1,4-diethynylbenzene. Yield: 21 mg (31%); ^{1}H NMR (300 MHz, $CDCl_3$): δ 7.57-7.55 (d, J = 8.4 Hz, 4H), 7.52 (s, 4H), 7.42-7.39 (d, J = 8.4 Hz, 4H), 2.44 (s, 6H); ^{13}C NMR (75.5 MHz, $CDCl_3$,): δ 193.4, 134.3, 132.2, 131.7, 128.4, 124.3, 123.0, 90.7, 90.6, 30.3; HRMS (ESI): *m/z* calcd for $C_{26}H_{19}O_2S_2$: 427.0821; found: 427.0812 ($M+H^+$).

Synthesis of M1. Following the similar synthetic procedure for ***m*-OPE**, 1,3-diethynylbenzene was replaced with 3,5-diethynylpyridine and the reaction mixture was stirred at 45 °C. Finally the resultant residue was purified by silica gel flash chromatography using a methanol/dichloromethane mixture (1/150 by volume) as eluent to afford the analytically pure product. Yield: 20 mg (30%); ^{1}H NMR (300 MHz, $CDCl_3$): δ 8.69 (s, 2H), 7.96-7.94 (t, *J* = 7.7 Hz, 4H), 7.59-7.56 (d, *J* = 8.4 Hz, 4H), 7.44-7.42 (d, *J* = 8.4 Hz, 4H), 2.44 (s, 6H); ^{13}C NMR (75.5 MHz, $CDCl_3$): δ 193.2, 140.8, 134.3, 132.3, 129.1, 123.4, 117.5,117.5, 92.7, 30.3; HRMS (ESI): *m/z* calcd for $C_{25}H_{18}NO_2S_2$: 428.0773; found: 428.0785 ($M+H^+$).

Synthesis of P. Following the same synthetic procedure for **M1**, 3,5-diethynylpyridine was replaced with 2,5-diethynylpyridine. The resultant residue was purified by silica gel flash chromatography using dichloromethane as eluent to produce the desired product. Yield: 27 mg (40%); ^{1}H NMR (300 MHz, $CDCl_3$): δ 8.77-8.76 (d, *J* = 1.1 Hz, 1H), 8.83-8.79 (dd, *J* = 2.0 Hz, *J* = 8.2 Hz, 1H), 7.64-7.62 (d, *J* = 8.3 Hz, 2H), 7.59-7.57 (d, *J* = 8.3 Hz, 2H), 7.54-7.51 (dd, *J* = 0.8 Hz, *J* = 8.4 Hz, 1H), 7.44-7.41 (d, *J* = 8.3 Hz, 4H), 2.45 (s, 6H); ^{13}C NMR (75.5 MHz, $CDCl_3$): δ 193.2, 193.1, 152.5, 141.8, 138.6, 134.3, 134.2, 132.6, 132.3, 129.4, 129.1, 126.6, 123.5, 123.2, 119.4, 93.7, 90.5, 89.9, 87.4, 30.3; HRMS (ESI): *m/z* calcd for $C_{25}H_{18}NO_2S_2$: 428.0773; found: 428.0762 ($M+H^+$).

Synthesis of compound M2. Following the same synthetic procedure for **M1**, 3,5-diethynylpyridine was replaced with of 2,4-diethynylpyridine and the reaction mixture was stirred at 35 °C. Finally the resultant residue was purified by silica gel flash chromatography using a methanol/dichloromethane mixture (1/100 by volume) as eluent to afford the desired product. Yield: 18 mg (27%); ^{1}H NMR (300 MHz, $CDCl_3$): δ 8.63-8.61 (d, *J* = 4.8 Hz, 1H), 7.65-7.57 (m, 5H), 7.45-7.44 (m, 4H), 7.35-7.33 (dd, *J* = 1.4 Hz, *J* = 5.0 Hz, 1H), 2.45 (s, 3H), 2.44 (s, 3H); ^{13}C NMR (75.5 MHz, $CDCl_3$): δ 193.2, 193.1, 150.1, 143.3, 134.3,134.2, 132.6, 132.5,131.8, 129.6, 129.3, 129.1, 124.7, 123.2, 123.0, 93.9, 89.5, 89.2, 87.5, 30.4, 30.3; HRMS (ESI): *m/z* calcd for $C_{25}H_{18}NO_2S_2$: 428.0773; found: 428.0765 ($M+H^+$)

Synthesis of compound M3. Following the same synthetic procedure for **M2**, 2,4-diethynylpyridine was replaced with 2,6-diethynylpyridine. Yield: 23 mg (35%); ^{1}H NMR (300 MHz, $CDCl_3$): δ 7.73-7.67 (m, 1H), 7.64-7.62 (d, *J* = 8.4 Hz, 4H), 7.51-7.49 (d, *J* = 7.8 Hz, 2H), 7.73-7.40 (d, *J* = 8.4 Hz, 4H), 2.44 (s, 6H); ^{13}C NMR (75.5

MHz, $CDCl_3$): δ 193.1, 143.5, 136.6, 134.2, 132.7, 129.3, 126.6, 123.2, 89.5, 89.0, 30.3; HRMS (ESI): *m/z* calcd for $C_{25}H_{18}NO_2S_2$: 428.0773; found: 428.0764 ($M+H^+$).

2. Computational methods

The Hamiltonian of the structures described in this paper were obtained using density functional theory as described below or constructed from a simple tight-binding model with a single orbital per atom of site energy $\varepsilon_0 = 0$ and nearest neighbour couplings $\gamma = -1$.

DFT calculation: The optimized geometry and ground state Hamiltonian and overlap matrix elements of each structure was self-consistently obtained using the SIESTA[10] implementation of density functional theory (DFT). SIESTA employs norm-conserving pseudo-potentials to account for the core electrons and linear combinations of atomic orbitals to construct the valence states. The generalized gradient approximation (GGA) of the exchange and correlation functional is used with the Perdew-Burke-Ernzerhof parameterization (PBE)[11] a double-ζ polarized (DZP) basis set, a real-space grid defined with an equivalent energy cut-off of 250 Ry. The geometry optimization for each structure is performed to the forces smaller than 40 meV/Ang.

3. Transport calculation

The mean-field Hamiltonian obtained from the converged DFT calculation or a simple tight-binding Hamiltonian was combined with our implementation of the non-equilibrium Green's function method, the GOLLUM,[12] to calculate the phase-coherent, elastic scattering properties of the each system consist of left (source) and right (drain) leads and the scattering region. The transmission coefficient *T(E)* for electrons of energy E (passing from the source to the drain) is calculated via the relation $T(E) = Trace(\Gamma_R(E)G^R(E)\Gamma_L(E)G^{R\dagger}(E))$. In this expression, $\Gamma_{L,R}(E) = i\left(\sum_{L,R}(E) - \sum_{L,R}{}^{\dagger}(E)\right)$ describe the level broadening due to the coupling between left (L) and right (R) electrodes and the central scattering region, $\sum_{L,R}(E)$ are the retarded self-energies associated with this coupling and $G^R = (ES - H - \sum_L - \sum_R)^{-1}$ is the retarded Green's function, where H is the Hamiltonian and S is overlap matrix. Using obtained transmission coefficient ($T(E)$), the conductance could be calculated by Landauer formula ($G = G_0 \int dE\, T(E)(-\partial f/\partial E)$) where $G_0 = 2e^2/h$ is the conductance quantum, $f(E) = (1 + \exp((E - E_F)/k_B T))^{-1}$ is the Fermi-Dirac

distribution function, T is the temperature and $k_B = 8.6x10^{-5}$ eV/K is Boltzmann's constant.

4. M-theory for heteroatoms

A key conceptual advance underpinned by the magic ratio rule (MRR)[13] is that it allows us to isolate deterministic quantum interference effects associated with the core of a molecule from the fluctuating contributions associated with random binding modalities, which inevitably occur in measurements of single-molecule conductance using techniques such as mechanically-controlled or STM break junctions. Here we outline the conceptual and mathematical steps which underpin the MRR and associated rules governing the heteroatom effect.

Step 1. The first concept is that of a "compound electrode", which is illustrated by Figure S1 below, in which the core of a molecule is connected *via* acetylene linkers to phenyl rings, which themselves are coupled *via* thiols to gold electrodes. For the molecules of chart 1 (main text) the central core is either a phenyl or a pyridyl ring.

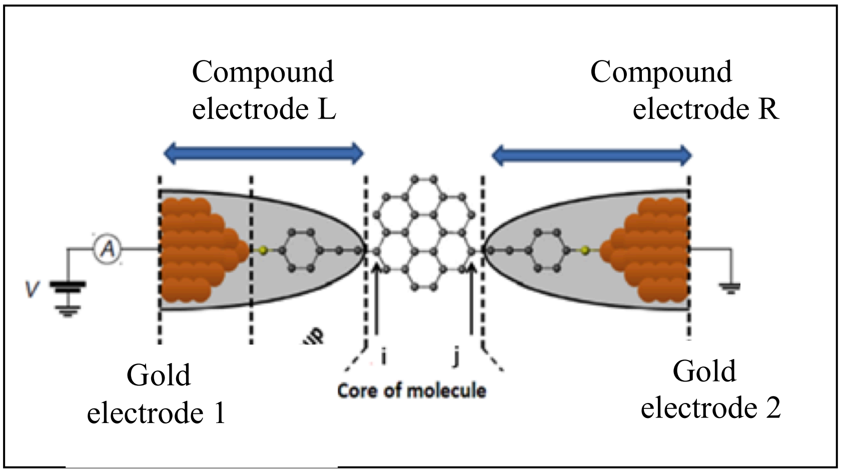

Figure S1 Sketch of a molecular junction illustrating the concept of compound electrodes.

One can view the above structure as either

(a) a simple molecular core, connected to complicated "compound electrodes" each formed from an acetylene linker, phenyl ring, thiol and gold electrode in series,

or alternatively as

(b) a simple gold electrode in contact with a complicated molecule formed from a central core connected *via* acetylene linkers to phenyl rings terminated by thiol groups.

Of course the transmission coefficient T(E) is independent of which viewpoint is adopted. However it turns out that because the acetylene groups form tunnel barriers, which act as weak links to the core, choice (a) simplifies the mathematical analysis of transport through such structures.

The following two mathematically equivalent expressions for T(E) capture these two viewpoints:

$$\mathrm{T(E)} = 4\mathrm{Tr}\,[\Gamma_2 G_{XX} \Gamma_1 \mathrm{G}_{XX}^{\dagger}] \quad (1)$$

and

$$\mathrm{T(E)} = 4\mathrm{Tr}\,[\Gamma_L G_{CC} \Gamma_R \mathrm{G}_{CC}^{\dagger}] \quad (2)$$

The latter is alternative notation for equation (11) in SI of the ref. 8b and equation (3) of the ref. 4d in the main text, whereas the former is commonly-presented form used in non-equilibrium Green's function transport codes, such as TRANSIESTA, TURBOMOL, SMEAGOL and GOLLUM.

In equation (1), Γ_2 and Γ_1 are level broadenings due to contact between the complicated molecule and gold leads labeled 1 and 2 and G_{XX} is the Green's function of the complicated molecule in the presence of such contacts. In contrast, in equation (2), Γ_L and Γ_R are level broadenings due to contact between the simple PAH core and the left and right compound electrodes L, R and G_{CC} is the Green's function of the simple core in the presence of such contacts.

Step 2. The two expressions (1) and (2) are mathematically equivalent, but when the coupling of the simple core to the compound electrodes is *via* weak acetylene linkers, equation (2) is preferred, because as noted in ref. 4g of the main text, provided the energy E does not coincide with an eigenvalue of the isolated simple core,

$$G_{CC} \approx g \quad (3)$$

where g is the Greens function of the isolated simple core. *ie* $g = (E - H)^{-1}$, where H is the Hamiltonian of the isolated core. If the *i,j* th element of g is g_{ij} and if the acetylene linkers connect only to site orbitals i and j of the core, then equation (2) yields for the transmission coefficient $\mathrm{T_{ij}(E)}$ corresponding to connectivity *i,j*

$$\mathrm{T_{ij}(E)} \approx \mathrm{B}_{LR} |g_{ij}(E)|^2 \quad (4)$$

where B_{LR} is independent of i and j.

Step 3. The quantity B_{LR} is a property of the electrodes and the coupling between the thiol group and the electrodes and therefore in a break junction, should be considered

a random quantity which fluctuates from measurement to measurement. In such experiments it is conventional to analyze histograms of the logarithm of the conductance. Therefore we are interested in the statistics of

$$\log \mathrm{T_{ij}(E)} \approx \log \mathrm{B}_{LR} + \log |g_{ij}(E)|^2$$

If the most probable value of $\ln \mathrm{T_{ij}(E)}$ is denoted $< \ln \mathrm{T_{ij}(E)} >$ then

$$< \log \mathrm{T_{ij}(E)} > \approx < \log \mathrm{B}_{LR} > + \log |g_{ij}(E)|^2$$

where $\log |g_{ij}(E)|^2$ is not subject to statistical variation.

If the statistical properties of $\ln \mathrm{B}_{LR}$ are independent of *i,j*, then when compared with two other connectivities *l,m*, we find

$$< \log \mathrm{T_{ij}(E)} > - < \log \mathrm{T_{lm}(E)} >$$
$$\approx [< \log \mathrm{B}_{LR} > + \log |g_{ij}(E)|^2] - [< \log \mathrm{B}_{LR} > + \log |g_{lm}(E)|^2]$$

ie $< \log \mathrm{T_{ij}(E)} > - < \log \mathrm{T_{lm}(E)} > \approx \log |g_{ij}(E)|^2 - \log |g_{lm}(E)|^2]$

Writing

$$\overline{\mathrm{T}}_{\mathrm{ij}}(\mathrm{E}) = 10^{<\log \mathrm{T_{ij}(E)}>} \qquad (5)$$

yields

$$\frac{\overline{\mathrm{T}}_{\mathrm{ij}}(\mathrm{E})}{\overline{\mathrm{T}}_{\mathrm{lm}}(\mathrm{E})} = \frac{|g_{ij}(E)|^2}{|g_{lm}(E)|^2} \qquad (6)$$

Assuming that T(E) does not vary significantly with energy on the scale of k_BT, we define the 'statistically-most-probable electrical conductance' σ_{ij} by $\sigma_{ij} = (\frac{2e^2}{h})\overline{\mathrm{T}}_{\mathrm{ij}}(\mathrm{E}_F)$ yields a general MRR:

$$\frac{\sigma_{\mathrm{ij}}}{\sigma_{\mathrm{lm}}} = \frac{|g_{ij}(\mathrm{E}_F)|^2}{|g_{lm}(\mathrm{E}_F)|^2} = \frac{\tau_{\mathrm{ij}}(\mathrm{E}_F)}{\tau_{\mathrm{lm}}(\mathrm{E}_F)} \qquad (7)$$

where we have defined the "core transmission coefficient" $\tau_{\mathrm{ij}}(\mathrm{E})$ by

$$\tau_{\mathrm{ij}}(\mathrm{E}) = |g_{ij}(E)|^2 \qquad (8)$$

Such core transmission coefficients are a useful concept, because they can be compared with DFT calculations of the full transmission coefficient T(E) and as shown in ref [8b] of the main text, are found to be qualitatively similar.

Equation (7) demonstrates that conductance ratios can be independent of the nature of the coupling between a molecule and the electrodes, even though the conductances themselves are not.

Step 4. To obtain the simplest description of conductance ratios, it is convenient to define the quantity

$$M(E) = A(E)g(E) \qquad (9)$$

Where $A(E)$ is a scalar function of energy. The simplest approximation to the pi-orbital Hamiltonian H of an isolated PAH core is $H = -C$, where apart from an overall constant which simply fixes the energy scale, C is a connectivity matrix, with entries $C_{ij} = 1$ if ij are nearest neighbours and zero otherwise. Consequently $A(0)$ can be chosen such that $M(0) = A(0)C^{-1}$ is simply a table of integers.

Step 5. For bipartite PAH cores, the middle of the HOMO-LUMO gap of such a Hamiltonian is $E = 0$ and therefore if E_F lies in the vicinity of the mid-gap, equation (7) reduces to the mid-gap ratio rule

$$\frac{\sigma_{ij}}{\sigma_{lm}} = \left[\frac{M_{ij}(0)}{M_{lm}(0)}\right]^2 \qquad (10)$$

Consequently the ratio of two conductances is simply the ratio of the squares of two integers.

For non-bipartite PAH cores such as azulene, $M(0)$ is also a table of integers. However even for the simple Hamiltonian $H = -C$, the energy corresponding to the middle of the HOMO-LUMO gap is no longer E=0. If the mid-gap energy is denoted E_{HL}, then if transport takes place in the middle of the HOMO-LUMO gap, equation (10) should be replaced by $\frac{\sigma_{ij}}{\sigma_{lm}} = \left[\frac{M_{ij}(E_{HL})}{M_{lm}(E_{MHL})}\right]^2$. Alternatively if E_F does not coincide with E_{HL}, then $\frac{\sigma_{ij}}{\sigma_{lm}} = \left[\frac{M_{ij}(E_F)}{M_{lm}(E_F)}\right]^2$.

Step 6. To verify the utility of the above concepts and series of approximations, the Table S1 (reproduced from *J. Am. Chem. Soc.* **2015**, *137*, 11425−11431), compares MRR predictions with experiment, for the ratios of conductances obtained using either the red or the blue connectivities. This demonstrates that although the MRR says nothing about the widths of conductance peaks and other statistical features, it does predict ratios of most the probable conductances quoted experimentally. It is worth mentioning that the MRR predictions below are based on an assumption of mid-gap transport, where the mid-gap energy E_{HL} is a property of the connectivity table C. Hence the MRR contains no free parameters. In contrast the predictions of GW theory and DFT shown in the table below are obtained only after treating the Fermi energy as a free parameter and adjusting it to obtain the best agreement with experiment.

Table S1. Comparison between the MRR and experiment.

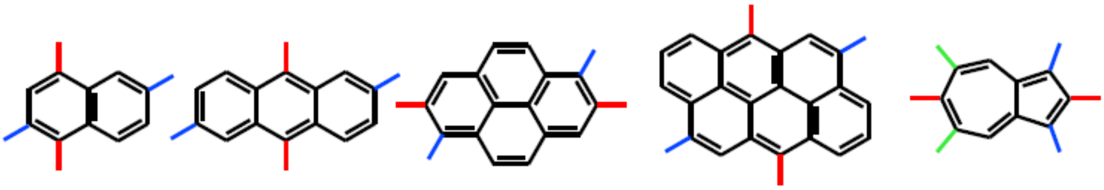

Molecular heart	Anchor group	Literature notation "Conductance Ratio of connectivity"	Mid-gap MRR	Experimental ratios	GW prediction	DFT Prediction
Naphthalene	thiol	Red / Blue (JACS, 2012)	4	5.1	NA	2
Anthracene	thiol	Red / Blue (JACS, 2012)	16	10.2	NA	13
Pyrene	carbon	Red / Blue (JACS, 2015)	9	8	NA	9
Anthanthrene	pyridyl	Red / Blue (JACS, 2015)	81	79	NA	81
Azulene	thiochroman	Red / Blue (Nano Lett., 2014)	0.72	1	0.32	0.93
Azulene	thiochroman	Green / Blue (Nano Lett., 2014)	0.003	0.06	0.1	0.05

Step 7. To understand the effect of introducing a heteroatom into a PAH core, it is useful to to regard the PAH core as a 'parent' and the heteroatom-substituted core as a 'daughter.'[14] In the presence of a heteroatom on site *l*, the parent Hamiltonian is modified by assigning a non-zero value ε to the diagonal element H_{ll}. As well as altering the Hamiltonian, charge transfer due to heteroatom substitution may cause the Fermi energy of the daughter (E_F^d) to differ from the Fermi energy (E_F^p) of the parent,

in which case when comparing electrical conductances of parents and daughters, equation 7 takes the more general form

$$\frac{\sigma_{ij}^{d}}{\sigma_{lm}^{p}} = \tau_{ij}^{d}(E_F^d)/\tau_{lm}^{p}(E_F^p) \qquad (11)$$

where $\tau_{ij}^{p}(E)$ and $\tau_{ij}^{d}(E)$ are core transmissions of the parent and daughter respectively.

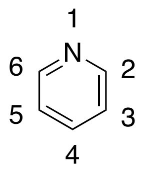

Figure S2 A convenient numbering of the central pyridine core. To retain consistency, the same numbering system is used for the parent benzene lattice.

Table S2 (a) The connectivity table C and (b) the M-table M(0) of a molecular core comprising a 6-membered ring. (c) The daughter Hamiltonian H^d in the presence of a heteroatom on site 1, with a nitrogen on-site energy ε. (d) The M-table $M^d(0)$ corresponding to H^d. (e) The M-table $M^d(E^d_{HL})$ corresponding to H^d, with ε = -0.5, where E^d_{HL} = -0.0795. (f) The M-table $M^d(E^d_F)$ corresponding to the optimal E^d_F = 0.211, with ε = -0.5.

C	1	3	5	2	4	6
1	0	0	0	-1	0	-1
3	0	0	0	-1	-1	0
5	0	0	0	0	-1	-1
2	-1	-1	0	0	0	0
4	0	-1	-1	0	0	0
6	-1	0	-1	0	0	0

a

$M^p(0)$	1	3	5	2	4	6
1	0	0	0	2	-2	2
3	0	0	0	2	2	-2
5	0	0	0	-2	2	2
2	2	2	-2	0	0	0
4	-2	2	2	0	0	0
6	2	-2	2	0	0	0

b

H^d	1	3	5	2	4	6
1	**ε**	0	0	-1	0	-1
3	0	0	0	-1	-1	0
5	0	0	0	0	-1	-1
2	-1	-1	0	0	0	0
4	0	-1	-1	0	0	0
6	-1	0	-1	0	0	0

c

$M^d(0)$	1	3	5	2	4	6
1	0	0	0	2	-2	2
3	0	0	0	2	2	-2
5	0	0	0	-2	2	2
2	2	2	-2	ε	-ε	ε
4	-2	2	2	-ε	ε	-ε
6	2	-2	2	ε	-ε	ε

d

$M^d(E^d_{HL})$	1	3	5	2	4	6
1	0.23	-0.08	-0.08	1.95	-1.96	1.95
3	-0.08	0.24	-0.08	2.03	1.99	-2.00
5	-0.08	-0.08	0.24	-2.00	1.99	2.03
2	1.95	2.03	-2.00	-0.25	0.41	-0.57
4	-1.96	1.99	1.99	0.41	-0.25	0.41
6	1.95	-2.00	2.03	-0.57	0.41	-0.25

e

$M^d(0.21)$	1	3	5	2	4	6
1	-0.72	0.24	0.24	2.26	-2.31	2.26
3	0.24	-0.67	0.22	2.01	2.13	-2.18
5	0.24	0.22	-0.67	-2.18	2.13	2.01
2	2.26	2.01	-2.18	-1.24	0.82	-0.36
4	-2.31	2.13	2.13	0.82	-1.27	0.82
6	2.26	-2.18	2.01	-0.36	0.82	-1.24

f

Table S2b demonstrates that for bipartite parental cores, in which odd-numbered sites are connected to even-numbered sites only, provided the Fermi energy of the daughter coincides with the mid-gap of the parent, the effect of heteroatom substitution onto an odd-numbered site is captured by the following rules, (which are easily obtained by solving Dysons equation in the presence of the site energy ε): (a) when *i* and *j* are odd, both parent and daughter have low conductances, (b) when *i* is odd and *j* is even, or *vice versa* both parent and daughter have high conductances and (c) when *i,j* are both even, the parent has a low conductance and the daughter a high conductance. When the daughter Fermi energy deviates from the mid-gap of the parent, Tables S2e and S2f reveal that these tendencies persist.

5. Comparison with DFT

Figures S3 shows the core transmission coefficients of the parent and daughter molecules shown in Chart 1, while Figure S4 shows DFT results for the full transmission coefficients. These are identical to the results of Figure 3 of the main text, but plotted over a wider energy range.

The SIESTA calculation was fully self consistent, including the Au contacts and the self energy (whose anti-hermitian contribution is the Gamma term) is included in the Gollum calculation. Consequently the energy shift due to the contacts is included in a fully self-consistent manner. In Figure 3B and S4, there is an extra low-conductance pathway due to the non-π system. This is only significant in cases where the conductance due to the π system is low, as in the case of **M1**. In the other M-OPE cases, as shown in Figure 3A, the π-system conductances are higher and therefore the contribution of the non-π system is masked.

It is well known that DFT does not accurately predict the energies of molecular orbitals relative to the Fermi energy of the electrodes. This issue is not confined to DFT. As mentioned above Table S1, this deficiency is also present in more sophisticated methods such as GW theory. Therefore when interpreting such calculations it is preferable to identify a more reliable reference energy. In Figure S3, when computing core transmission coefficients from a Huckel Hamiltonian, we use the mid-gap of the parent as a reference energy. This choice is reasonable because the parent possesses particle-hole symmetry and in the case of meta connectivity, it is equivalent to choosing the energy at which destructive interference occurs. Therefore

to aid comparison with DFT, in Figure S4, T(E) is plotted only for a range of energies centred approximately on the middle of the parental HOMO-LUMO gap.

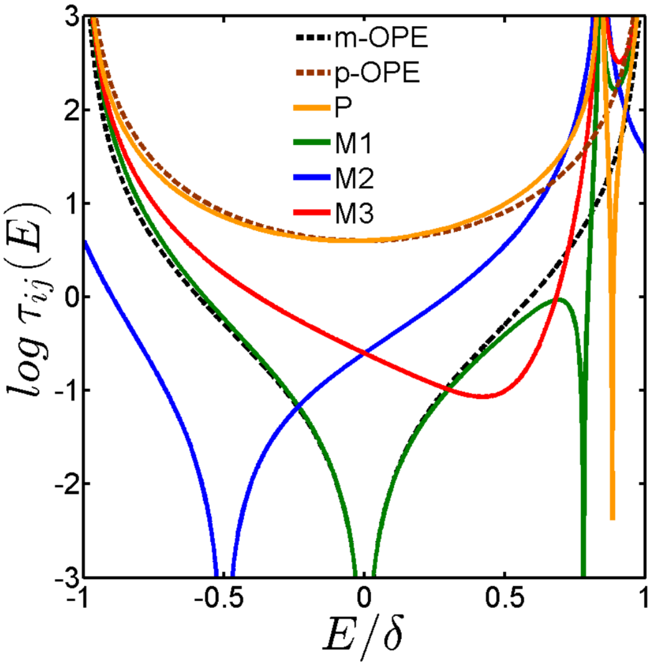

Figure S3 Core transmissions of the parent and daughter molecules shown in Chart 1 (with the exception of reference compounds **R1**-**R3**)

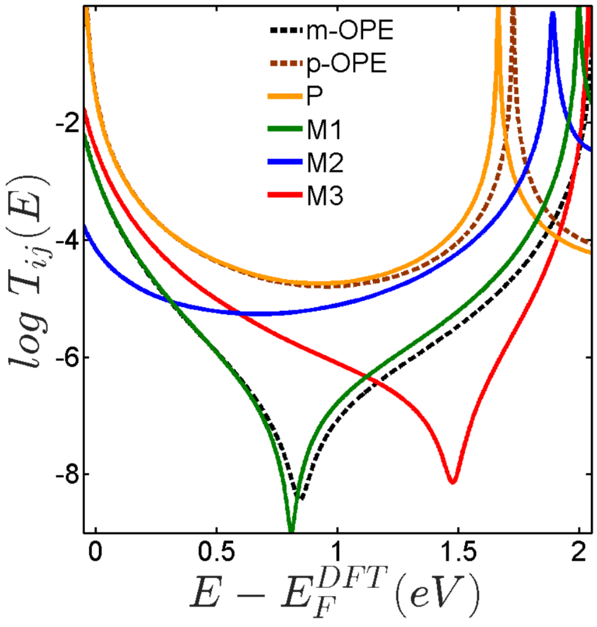

Figure S4 *DFT*-Gollum results for the electron transmission coefficients of junctions formed from the parent and daughter molecules shown in Figure S5.

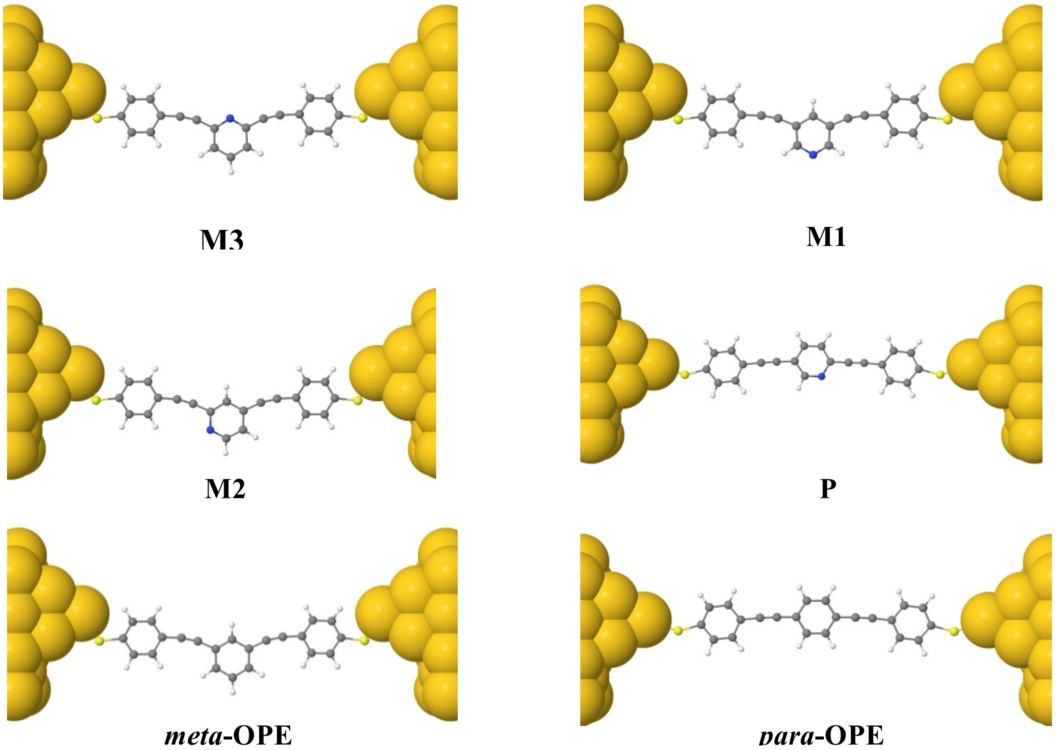

Figure S5 Relaxed molecular structures attached to the Au leads.

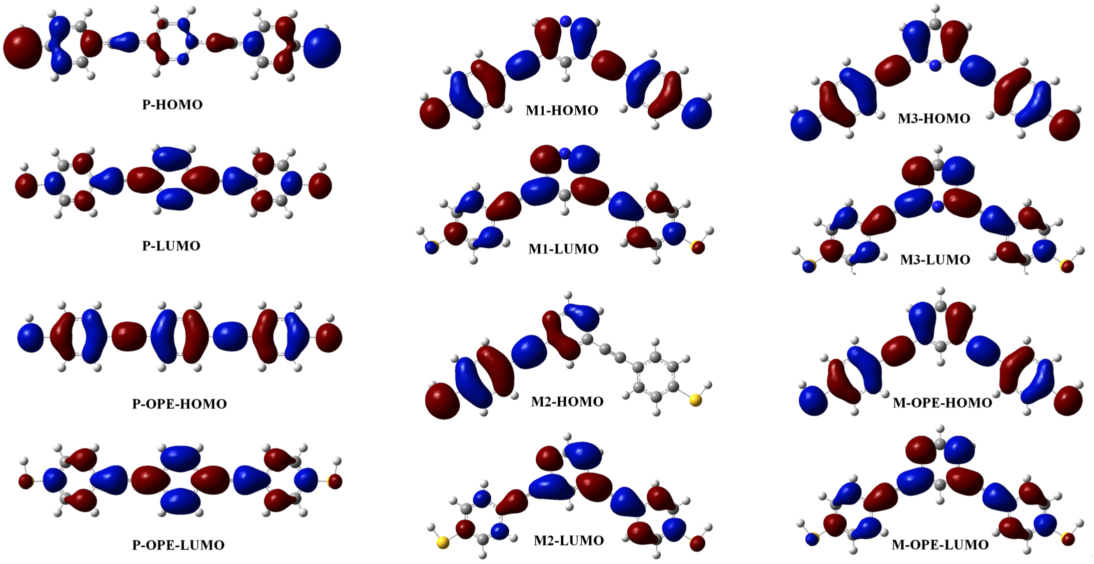

Figure S6 Frontier molecular orbitals of the molecules shown in Chart 1 with the exception of the reference compounds **R1**-**R3**.

6. Exclusion of alternative binding configurations

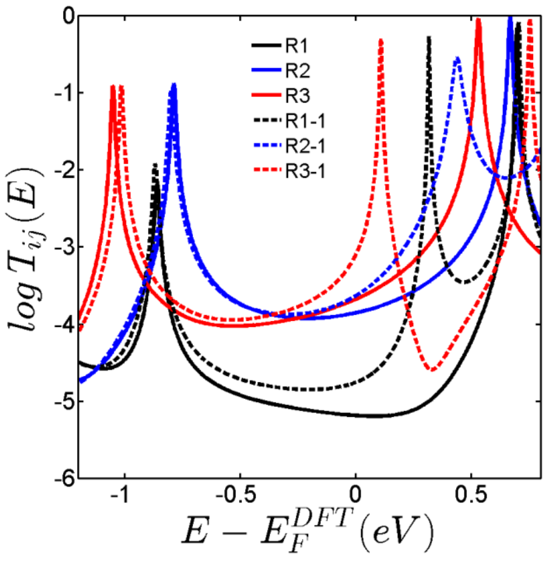

Figure S7 *DFT-NEGF* results for the electron transmission coefficients of junctions formed from molecules shown in Figure S8.

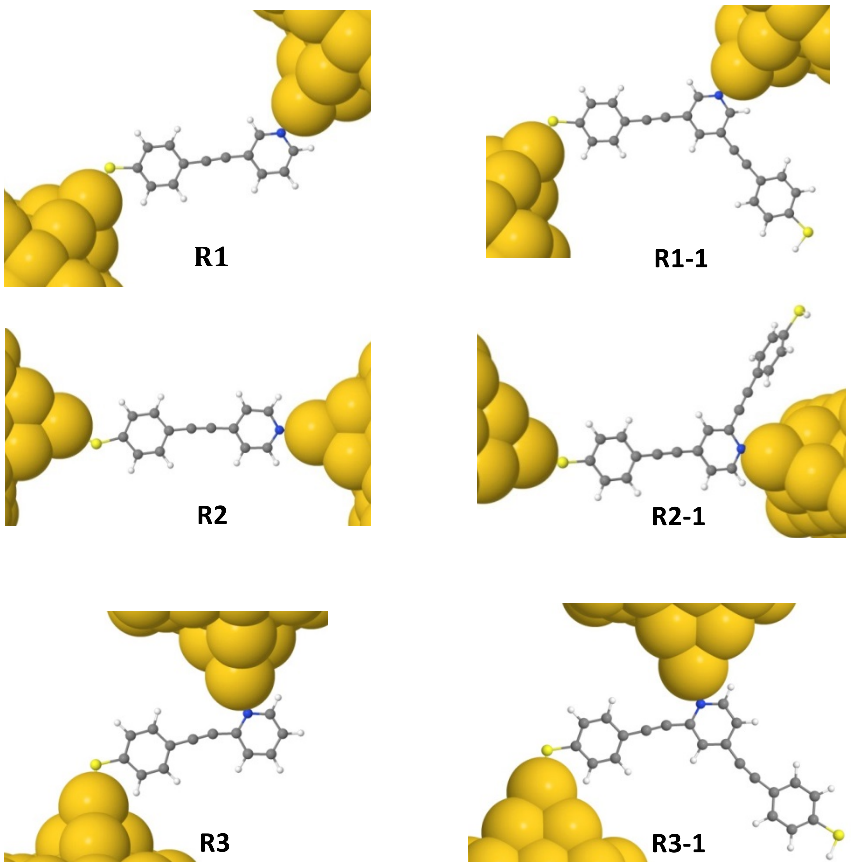

Figure S8 Relaxed molecular structures attached to the Au leads.

7. Molecular lengths and conductance histograms

Table S3 Calculated molecular lengths of **R1**-**R3** in different configurations. Au-Au distance is the distance between apex gold atoms of the electrodes.

	N-S/nm-experiment	N-S/nm theory	Au-Au/nm theory
**R1**	1.2	1.09	1.48
**R2**	1.3	1.16	1.63
**R3**	1.1	0.96	1.176

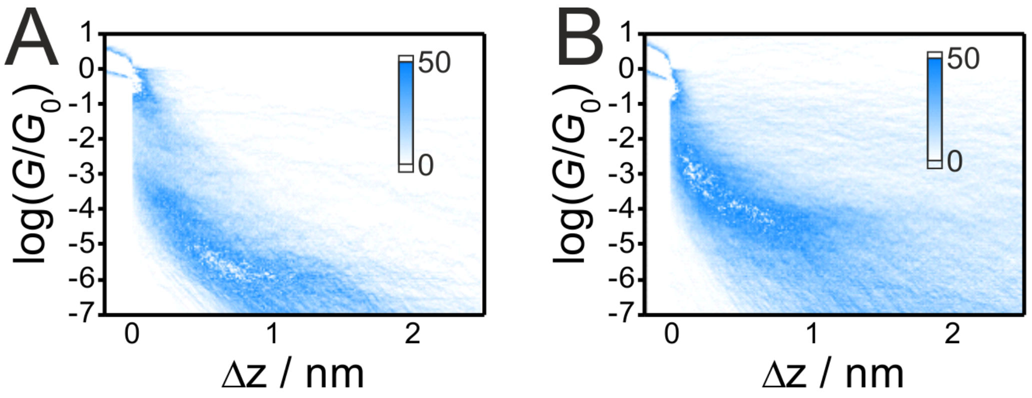

Figure S9 2D conductance histograms (A) of **M1** and (B) of **M2**.

8. NMR spectra

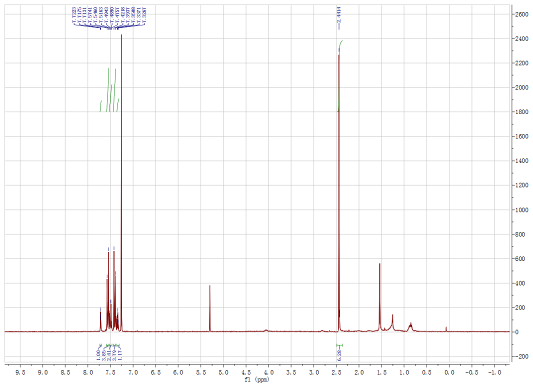

^{1}H NMR spectrum for compounds ***m*-OPE**

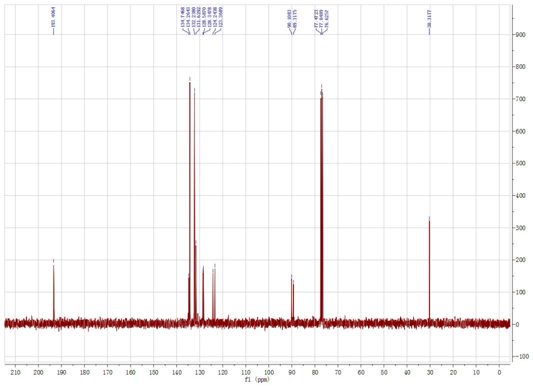

^{13}C NMR spectrum for compounds ***m*-OPE**

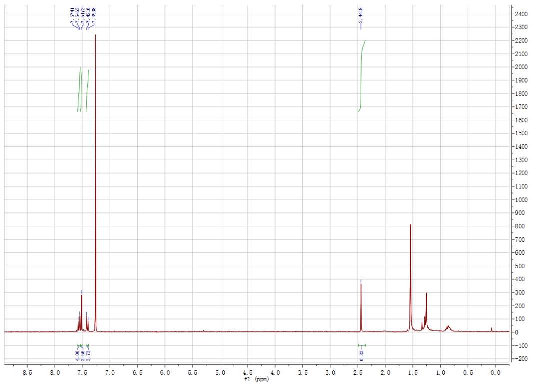

^{1}H NMR spectrum for compounds ***p*-OPE**

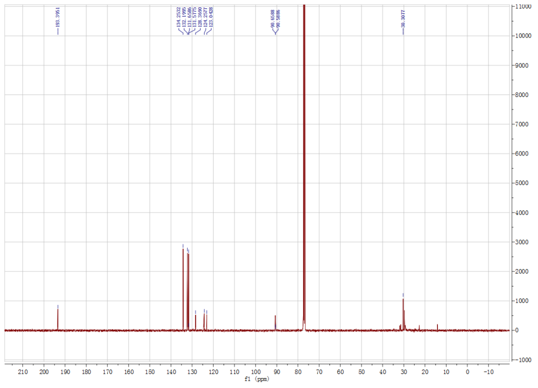

^{13}C NMR spectrum for compounds ***p*-OPE**

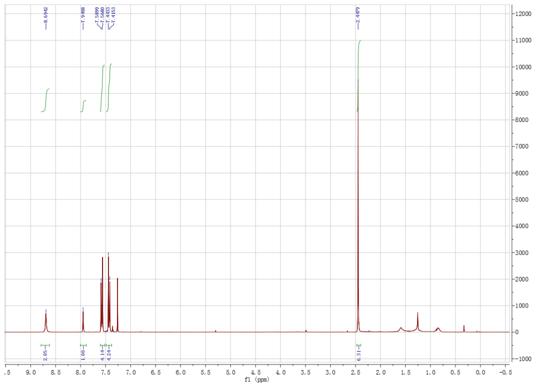

^{1}H NMR spectrum for compounds **M1**

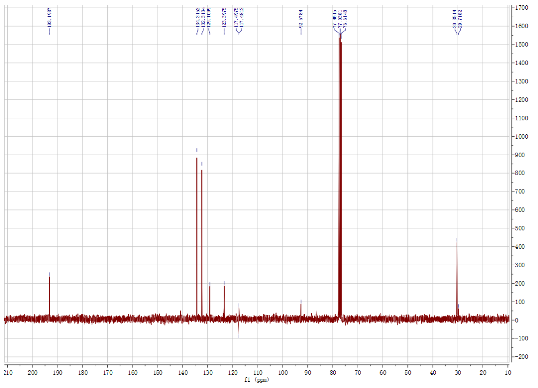

^{13}C NMR spectrum for compounds **M1**

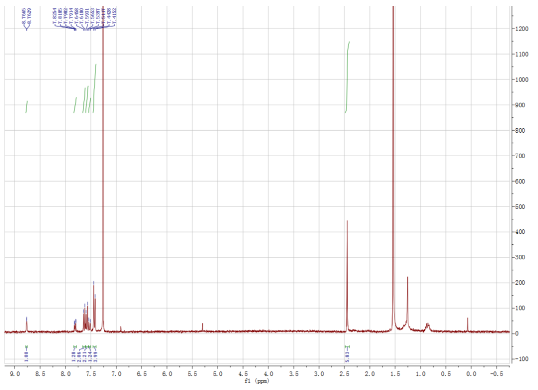

^{1}H NMR spectrum for compounds **P**

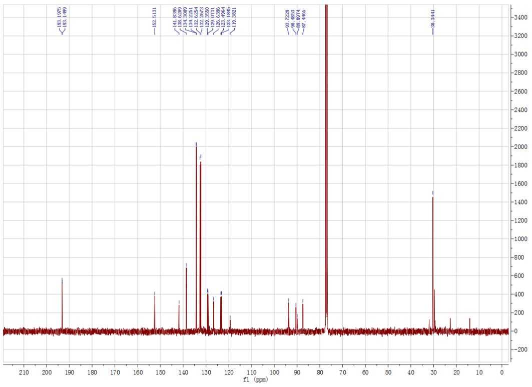

^{13}C NMR spectrum for compounds **P**

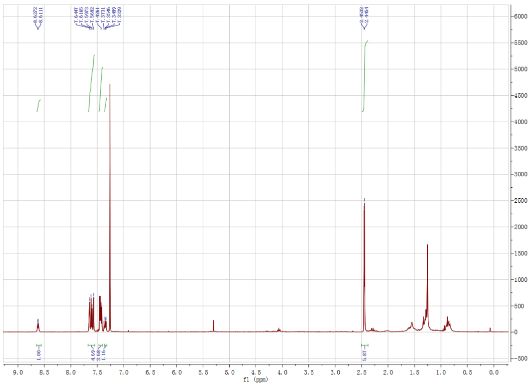

^{1}H NMR spectrum for compounds **M2**

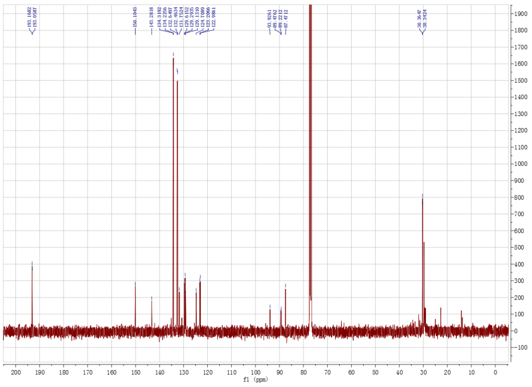

^{13}C NMR spectrum for compounds **M2**

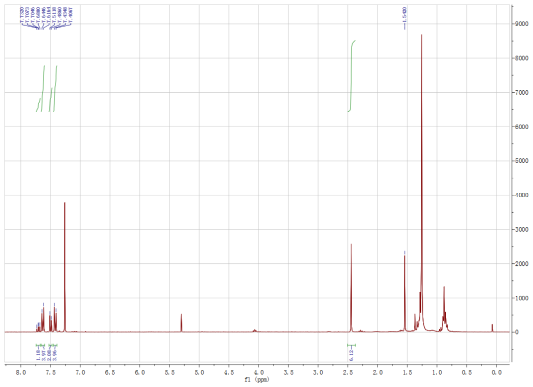

^{1}H NMR spectrum for compounds **M3**

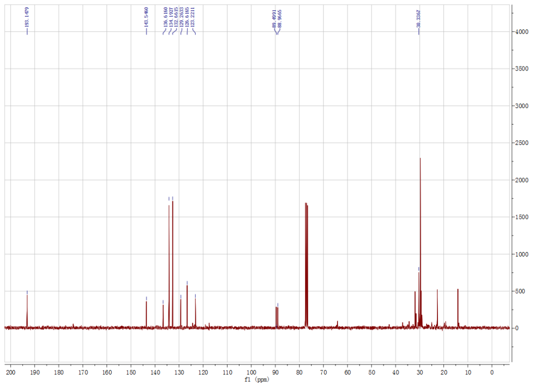

^{13}C NMR spectrum for compounds **M3**

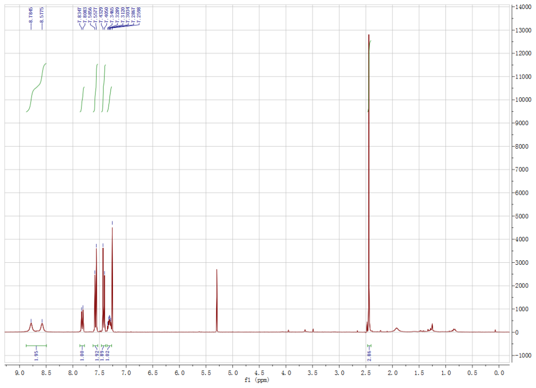

^{1}H NMR spectrum for compounds **R1**

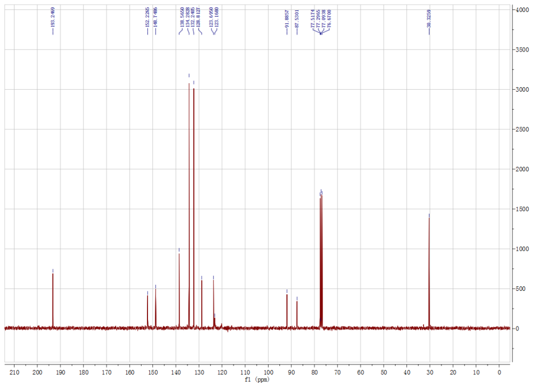

^{13}C NMR spectrum for compounds **R1**

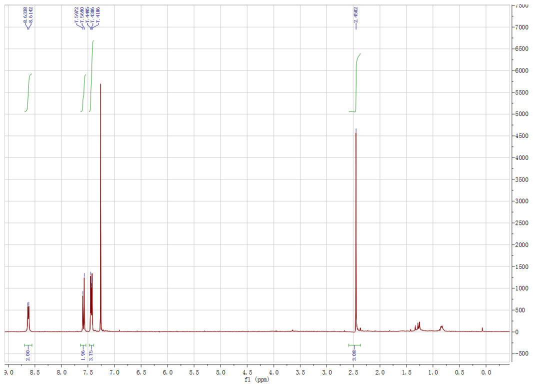

^{1}H NMR spectrum for compounds **R2**

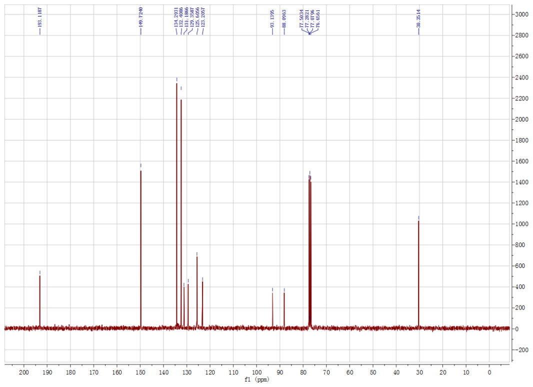

^{13}C NMR spectrum for compounds **R2**

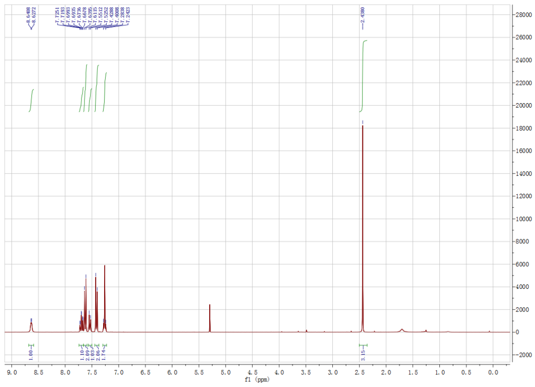

^{1}H NMR spectrum for compounds **R3**

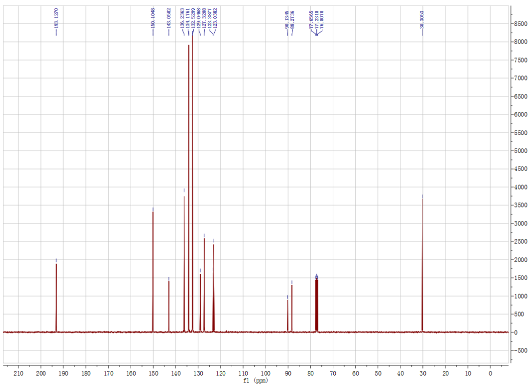

^{13}C NMR spectrum for compounds **R3**

References

[1] B. E. Bode, D. Margraf, J. Plackmeyer, G. Dürner, T. F. Prisner, O. Schiemann, *J. Am. Chem. Soc.* **2007**, *129*, 6736-6745.

[2] F. Giacalone, M. A. Herranz, L. Gruter, M. T. Gonzalez, M. Calame, C. Schonenberger, C. R. Arroyo, G. Rubio-Bollinger, M. Velez, N. Agrait, N. Martin, *Chem. Commun.* **2007**, 4854-4856.

[3] K. Liu, X. Wang, F. Wang, *ACS Nano* **2008**, *2*, 2315-2323.

[4] H. Goto, J. M. Heemstra, D. J. Hill, J. S. Moore, *Org. Lett.* **2004**, *6*, 889-892.

[5] J. Feng, C. Zhang, Y. Li, M. Yang, *J. Appl. Polym. Sci.* **2011**, *121*, 217-225.

[6] Y. Shin, G. E. Fryxell, C. A. Johnson Ii, M. M. Haley, *Chem. Mater.* **2008**, *20*, 981-986.

[7] M. A. Bartucci, J. W. Ciszek, *J. Org. Chem.* **2014**, *79*, 5586-5594.

[8] M. A. Bartucci, P. M. Wierzbicki, C. Gwengo, S. Shajan, S. H. Hussain, J. W. Ciszek, *Tetrahedron Lett.* **2010**, *51*, 6839-6842.

[9] J. M. Tour, A. M. Rawlett, M. Kozaki, Y. Yao, R. C. Jagessar, S. M. Dirk, D. W. Price, M. A. Reed, C.-W. Zhou, J. Chen, W. Wang, I. Campbell, *Chem. Eur. J.* **2001**, *7*, 5118-5134.

[10] J. M. Soler, E. Artacho, J. D. Gale, A. Garcia, J. Junquera, P. Ordejon, D. Sanchez-Portal, *J. Phys.: Condens. Matter* **2002**, *14*, 2745-2779.

[11] J. P. Perdew, K. Burke, M. Ernzerhof, *Phys. Rev. Lett.* **1996**, *77*, 3865-3868.

[12] J. Ferrer, C. J. Lambert, V. M. García-Suárez, D. Z. Manrique, D. Visontai, L. Oroszlany, R. Rodríguez-Ferradás, I. Grace, S. W. D. Bailey, K. Gillemot, S. Hatef, L. A. Algharagholy, *New J. Phys.* **2014**, *16*, 093029.

[13] a) S. Sangtarash, C. Huang, H. Sadeghi, G. Sorohhov, J. Hauser, T. Wandlowski, W. Hong, S. Decurtins, S.-X. Liu, C. J. Lambert, *J. Am. Chem. Soc.* **2015**, *137*, 11425-11431; b) Y. Geng, S. Sangtarash, C. Huang, H. Sadeghi, Y. Fu, W. Hong, T. Wandlowski, S. Decurtins, C. J. Lambert, S.-X. Liu, *J. Am. Chem. Soc.* **2015**, *137*, 4469-4476.

[14] S. Sangtarash, H. Sadeghi, C. J. Lambert, *Nanoscale* **2016**, *8*, 13199-13205.
